# The benefit of amplification on auditory working memory function in middle-aged and young-older hearing impaired adults

**DOI:** 10.3389/fpsyg.2015.00721

**Published:** 2015-06-05

**Authors:** Karen A. Doherty, Jamie L. Desjardins

**Affiliations:** ^1^Department of Communication Sciences and Disorders, Syracuse University, Syracuse, NY, USA; ^2^Department of Rehabilitation Sciences, University of Texas at El Paso, El Paso, TX, USA

**Keywords:** age-related hearing loss, presbycusis, aging, hearing aids, working memory

## Abstract

Untreated hearing loss can interfere with an individual’s cognitive abilities and intellectual function. Specifically, hearing loss has been shown to negatively impact working memory function, which is important for speech understanding, especially in difficult or noisy listening conditions. The purpose of the present study was to assess the effect of hearing aid use on auditory working memory function in middle-aged and young-older adults with mild to moderate sensorineural hearing loss. Participants completed two objective measures of auditory working memory in aided and unaided listening conditions. An aged matched control group followed the same experimental protocol except they were not fit with hearing aids. All participants’ aided scores on the auditory working memory tests were significantly improved while wearing hearing aids. Thus, hearing aids worn during the early stages of an age-related hearing loss can improve a person’s performance on auditory working memory tests.

## Introduction

Age-related hearing loss in middle-aged (MA) and young-older (YO) adults is a public health problem in the U.S. affecting 20% of people between 45–59 years of age and 33% of people in their sixties (National Institute on Deafness and Other Communication Disorders, 2012; Cruickshanks et al., 2003; Nash et al., 2011). Age-related hearing loss is initiated peripherally in the auditory system, and involves hair cell loss, a decline in the cochlear metabolic system, and a loss of spiral ganglion neurons (Frisina and Walton, 2001). The peripheral loss begins in the high frequency regions of the peripheral auditory system and projects to the high frequency regions of the brain, which can induce reorganization of auditory cortical frequency maps (Robertson and Irvine, 1989; Harrison et al., 1991). Due to its gradual onset, mild age-related hearing loss often goes unnoticed. Although signs for early hearing loss exist, many people are unaware of them or choose not to acknowledge them. Instead, they will place the onus of their communication problems on others. For example, individuals with hearing impairment will often suggest people mumble, do not speak clearly, or speak too softly.

Currently, hearing aids are the primary treatment for an age-related hearing loss. However, the uptake rates for adult hearing aid use are low; 20% for all hearing impaired adults, and 15% for adults with hearing loss in their fifties (Lin, 2011). Furthermore, on average, it takes individuals about 10 years from the time they become aware of their hearing problems to when they seek treatment (Davis et al., 2007). This is concerning because age-related hearing loss can be a serious communication disorder that when left untreated can negatively impact a person’s social, and psychological function (for a review, see National Council on the Aging, 1999). Untreated hearing loss has also been related to cognitive function (Lindenberger and Baltes, 1994a; Pichora-Fuller and Singh, 2006; Arlinger et al., 2009; Lin et al., 2013). For example, Lindenberger and Baltes (1994b) found that peripheral auditory thresholds were significantly related to processing speed, working memory, and reasoning in 156 individuals who were 70 years and older. Similarly, van Boxtel et al. (2000) reported a significant relationship between auditory function and verbal memory performance in 453 individuals between 23 and 82 years of age. Recently, Desjardins and Doherty (2013) found that working memory, processing speed and selective attention abilities were significantly associated with older hearing impaired adults’ speech recognition performance in background noise.

It has been suggested that a lack of auditory input from an untreated hearing loss could negatively affect the neural networks involved in certain cognitive abilities (Sekuler and Blake, 1987; Lindenberger and Baltes, 1994a; Belin et al., 1999; Wong et al., 2010). That is, a perceptual decline could result in a permanent cognitive decline (*deprivation hypothesis*, Baltes and Lindenberger, 1997). It has also been suggested that even mild hearing loss could lead to a decline in cognitive performance because the cognitive resources normally used for higher-level comprehension, like storing auditory information into memory, must be used by the individual to accurately decode and perceive the speech signal (Pichora-Fuller and Singh, 2006; Rönnberg et al., 2008, 2013; Tun et al., 2009; Gosselin and Gagne, 2011; Desjardins and Doherty, 2013, 2014).

Fortunately, there is evidence that hearing aid use may improve older adults’ performance on auditorily presented cognitive tests because the amplified signal likely improves an individual’s perception of instructions and test items (Mulrow et al., 1990; Allen et al., 2003; and Weinstein and Amsel, 1986). Allen et al. (2003) reported reduced rates of decline in cognitive screening scores for dementia over a 6-month period following intervention with hearing aids in a group of older adults. Mulrow et al. (1990) found improved performance with the use of hearing aids on a general cognitive measure in adults in their 70 s with moderate sensorineural hearing loss. In addition, a group of older people with dementia were reclassified to a less severe category of dementia when retested with amplification (Weinstein and Amsel, 1986). Hearing aid use has also been shown to reduce listening effort on a speech recognition in noise listening task (Desjardins and Doherty, 2013, 2014). However, other studies have shown that hearing aid use had no effect on older hearing impaired listeners’ performance on visual measures of working memory and executive function (Tesch-Romer, 1997; van Hooren et al., 2005). Thus, studies that have examined the effects of hearing aid use on cognition have yielded different results.

In the present study, we examined the effect of hearing aid use on auditory tests of working memory in MA and YO adults. Despite the interest in the association between hearing impairment and cognitive function, only a few studies have investigated whether use of amplification improves working memory performance (Tesch-Romer, 1997; van Hooren et al., 2005). In addition, much of what we know about the negative effects of untreated hearing loss and the potential benefit of hearing aids to offset these effects is based on studies that include participants with an average age of 70 years leaving the earlier stages of age-related hearing loss less understood. We specifically chose to assess working memory function in this study because it has been shown to be necessary for effective speech-communication in noise (Baddeley and Hitch, 1974; Gatehouse et al., 2003; Humes et al., 2006; Akeroyd, 2008), and to decline with increasing age (Salthouse and Lichty, 1985).

Briefly, working memory is a system for the temporary storage, management, and manipulation of information required for carrying out complex cognitive tasks such as language comprehension (Daneman and Carpenter, 1980). Models of working memory assume that when the capacity limits of working memory are exceeded due to processing demands (e.g., background noise), either comprehension will become slowed or errors will occur (Rabbitt, 1990; Rönnberg, 2003). Thus, an impoverished perceptual input due to background noise or hearing impairment could compromise cognitive performance (Rabbitt, 1968; Lindenberger and Baltes, 1994a; Pichora-Fuller, 2008; Rönnberg et al., 2008, 2013). According to the ease of language understanding model (ELU; Rönnberg et al., 2013), in effort demanding listening situations (e.g., listening to speech in background noise), an individual with a high working memory capacity will be better able to compensate for a distorted signal without exhausting their working memory capacity (i.e., making listening less effortful), compared to an individual with a smaller working memory capacity (Rudner et al., 2011; Ng et al., 2013; Mishra et al., 2014; Rudner and Lunner, 2014). Thus, hearing aids may lessen the cognitive processing resources a hearing impaired listener must expend to understand speech by effectively compensating for an auditory impairment (Desjardins and Doherty, 2014).

## Materials and Methods

### Participants

**FIGURE 1:**
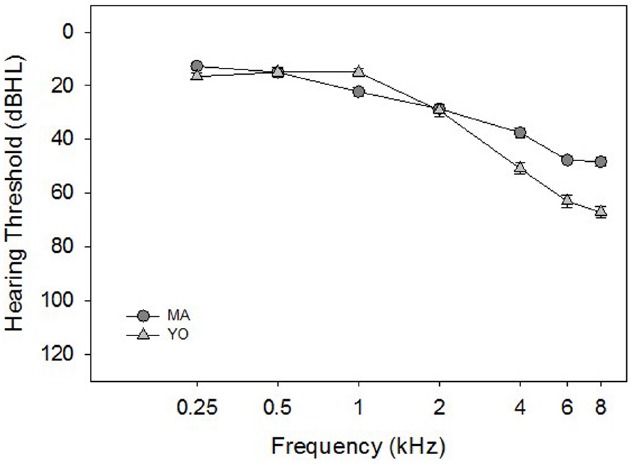
**Mean pure-tone thresholds (in dB HL) averaged across the right and left ears for the MA (circles), and YO (triangles) participants.** Error bars represent ± 1 SD.

There were 24 participants divided among 11 MA adults 50–60 years of age [Mean (M) = 56.6 years, Standard Deviation (SD) = 3.4 years], and 13 YO adults 63–74 years of age (*M* = 68.7 years, SD = 4.1 years). All of the participants in the current study were part of a larger longitudinal hearing aid study. All participants had at least a mild sensorineural hearing loss, bilaterally (i.e., two out of three thresholds were > 26 dB at 2 kHz, > 30 dB at 3 kHz and/or > 35 dB at 4 kHz), and no more than a 15 dB difference in hearing thresholds between ears at any audiometric frequency. This hearing loss criterion was selected so that the participants’ thresholds would be at least > 0.5 standard deviations from the normal hearing thresholds reported for these ages in the Cruickshanks et al. (2003) study. Mean pure-tone thresholds for the MA and YO participants averaged across the left and right ears are shown in Figure 1.

Two age-matched control groups were also included in this study. The purpose of the control participants was to ensure that any significant changes measured in the experimental groups over the 6 week study period were not a result of normal test re-test variability on the experimental test measures. The control groups consisted of a group of 8 MA (C-MA) (Mean Age = 55 years, SD = 2.9 years) adults, and a group of 8 YO (C-YO) (Mean Age = 67 years, SD = 3.1 years) adults. The control participants were recruited in the same manner as the experimental participants in this study. If a participant did not meet the hearing threshold criteria for being fit with hearing aids, they were assigned to one of the control groups depending upon their age.

None of the participants had worn or tried a hearing aid prior to participating in this study. All participants were native speakers of English and were paid an hourly wage for their participation. Institutional Review Board approval was obtained prior to commencement of this study in accordance with the Syracuse University IRB committee.

### Amplification

Middle-aged and YO participants were fitted with ReSound Alera 9 (GN ReSound, Ballerup Denmark) receiver-in-the-canal hearing aids coupled to open dome ear molds, bilaterally. Hearing aid gain was determined based on the Desired Sensation Level (DSL v. 5) prescriptive method (Scollie et al., 2005). DSL targets were generated using Avanti 3.2 software in NOAH 3, and verified with the Audio scan Verifit VF-1 real ear system (Dorchester, ON, Canada). The frequency responses of the hearing aids were adjusted so that the real-ear aided response was within 5 dB across the prescribed target values for 0.25, 0.5, 1, 2 kHz, and within 10 dB for 4 kHz and 6 kHz at an input signal of 70 dB SPL. The hearing aids were set to have two programs: (1) Omnidirectional, (2) Adaptive noise reduction. All other programs and the volume control were disabled. Participants were instructed on the use and care of their hearing aids, and asked to wear the hearing aids for at least 8 h per day, every day, for 6 weeks.

The data-logging feature in the hearing aids was used to track the overall hours of hearing aid use over the 6 week hearing aid trial. The Practical Hearing Aid Skills Test Revised (PHAST-R; Desjardins and Doherty, 2009; Doherty and Desjardins, 2012), an eight item objective assessment that measures basic hearing aid use and care skills, was administered to participants at their initial hearing aid fitting session, after 2 weeks of hearing aid use, and at 6 weeks of hearing aid use. The PHAST-R provided an objective measure of the participant’s ability to correctly use and care for their hearing aids. After each administration of the PHAST-R, participants were reinstructed on tasks they did not perform correctly or know how to perform.

### Test Measures

Working memory function was measured using an auditory version of The Reading Span Test (Daneman and Carpenter, 1980; Pichora-Fuller et al., 1995), and an auditory version of the *n*-back task (*N*-backer; Monk et al., 2011).

#### Listening Span Test

The Listening Span Test, which is an auditory version of the Reading Span Test, was selected to measure working memory because the Reading Span Test has been shown to be one of the best predictors of speech recognition performance in noise in hearing impaired adults (Akeroyd, 2008; Rönnberg et al., 2010; Desjardins and Doherty, 2013; Ng et al., 2013). The methods used to administer the Listening Span Test in the current study have methodological similarities to those reported for the auditory reading span test in previous studies (Pichora-Fuller et al., 1995; Sarampalis et al., 2009; Ng et al., 2013, 2015). The Listening Span Test in the present study consists of sentences from the revised Speech Perception in Noise (R-SPIN) test (Bilger et al., 1984) which is comprised of eight lists of 50 sentences (400 total sentences). Each list of sentences contains 25 high context sentences such that the final-word in the sentence is predictable (e.g., A chimpanzee is an ape) and 25 low context sentences where the final-word is not predictable (e.g., She might have discussed the ape). R-SPIN sentences were recorded by a female talker and digitized using the Computerized Speech Lab (Kay Elemetrics, Montvale, NJ, USA) at a 44,100 Hz sampling rate. They were presented at 70 dBSPL in quiet, and in a speech shaped noise (SSN) at +8 dB signal-to-noise-ratio (SNR). The SSN was generated in MATLAB using a 16 bit, 44. 1 kHz sampling rate, by passing a Gaussian noise through a Finite Impulse Response filter with a magnitude response equal to the Long Term Average Speech Spectrum of the 400 R-SPIN sentences. The +8 dB SNR level was chosen to avoid ceiling and floor effects on speech recognition performance based on pilot data we collected with MA and YO hearing impaired adults.

The R-SPIN sentences were presented to participants in a double walled sound attenuating booth in quiet and in the SSN in a randomized order via a Sony multi-disc CD changer (Sony electronics Inc., Tokyo, Japan) routed through a GSI-61 audiometer to a GSI loudspeaker (Grason-Stadler, Eden Prairie, MN, USA) located 1 meter, at ear level, in front of the participant (0°azimuth). In the SSN condition, the background masker was played continuously throughout the task. Participants were required to repeat the entire R-SPIN sentence they heard during a 4 s interval that followed the presentation of each sentence, and to remember the final word in each sentence for later recall. The examiner recorded only the final key word in the sentence. The memory task was manipulated by varying the number of sentences in the set (i.e., 2, 4, and 6). After all the sentences in a given set were presented, the experimenter prompted the participant to recall as many of the previously reported final key words as they could, verbally, and in any order. Twenty-four sentences were presented in each of the six experimental conditions (Quiet: set size 2, 4, 6, and Noise: set size 2, 4, 6). Performance on the Reading Span test was computed based on the percent of correctly recalled final key words.

#### *N*-back Test

Participants were administered an auditory version of the *n*-back task (*N*-backer; Monk et al., 2011). The *n*-back is a continuous performance task that is commonly used as an assessment in cognitive neuroscience to measure the executive component of working memory (for reviews, see Kane et al., 2007; Jaeggi et al., 2010). Participants were seated in a double-walled sound attenuating booth and presented a sequence of 25 randomly generated synthesized digits from 1 to 9 using the *N*-backer computer software (Monk et al., 2011) via a computer routed through a GSI-61 audiometer to a GSI loudspeaker (Grason-Stadler, Eden Prairie, MN, USA) located 1 meter, at ear level, in front of the participant (0°azimuth). Each digit was presented with a constant inter-stimulus interval of 2000 ms at 70 dBSPL in quiet and in a SSN at +8 dB SNR in a randomized order. In the SSN condition, the background masker was played continuously throughout the task. The +8 dB SNR level was chosen to avoid ceiling and floor effects on speech recognition performance based on pilot data we collected with this population. Participants were instructed to listen to the stream of randomly presented digits, and to say the digit they heard “1-step” back in time for the 1-back task, and to say the digit they heard “2-steps” back in time for the 2-back task. Participants always completed a practice test session first, during which streams of 10 randomly presented digits were presented in quiet and in noise. Performance on the auditory *n*-back was calculated as the number of correctly recalled digits.

### Procedure

Middle-aged and YO participants completed four test sessions over a period of 6 weeks. On the weeks when participants were not seen in the lab, they were contacted via telephone by the examiner to encourage hearing aid use, answer questions, and trouble shoot hearing aid problems. During session 1, all testing was performed unaided, hearing thresholds were obtained at the standard audiometric test frequencies from 0.25 to 8.0 kHz with a GSI-61 audiometer using standard audiometric test procedures (American National Standards Institute [ANSI], 2003). All stimuli were presented at 70 dB SPL, which was above the participants’ hearing thresholds. To further ensure that the stimuli were audibile we obtained speech recognition scores for the R-SPIN sentences and the *N*-back digits unaided in quiet and background noise. The Listening Span test and the auditory *N*-back were then administered in quiet and in noise in a randomized order. Session 2 took place within 1 week of session 1. During session 2, the experimental participants were fitted with hearing aids following the hearing aid fitting procedure described in the amplification section. Two weeks after their initial hearing aid fitting, participants returned to the lab to participate in Session 3. During session 3, hearing aid orientation information was reviewed. The PHAST-R (Doherty and Desjardins, 2012) was administered, and participants were reinstructed on the hearing aid use and care skills they did not perform correctly or know how to perform. In addition, participants aided speech recognition in quiet and noise was measured using lists of 24 sentences from the R-SPIN following the standard R-SPIN test instructions (see Bilger et al., 1984). After wearing the hearing aids for 6 weeks, participants returned to the lab for session 4. During session 4, participants were administered the Listening Span Test and the auditory *N*-back while wearing their hearing aids. All testing in background noise was performed with the hearing aids in the adaptive noise reduction setting. At the end of Session 4 participants were asked to return the hearing aids. Participants were then administered the auditory *n*-back test unaided.

The two age-matched control groups followed a similar testing procedure as the two experimental groups, except they were not fitted with amplification. Control participants completed Session 1, as described for the experimental participants. Six weeks after they completed session 1, they returned to the lab for a second test session (i.e., control-session 2). During Control-session 2, control participants were administered the Listening Span Test and the auditory *N*-back in a randomized order.

## Results

Speech recognition scores were compared across the control and experimental groups. The mean unaided sentence recognition (R-SPIN) scores were 98% (SD = 4), 95% (SD = 11), 100% (0), and 100% (SD = 0) in quiet and 98% (SD = 3), 94% (SD = 12), 100% (SD = 0), and 100% (SD = 0) in background noise for the MA, YO, C-MA and C-YO groups, respectively. Based on the 95% critical differences for speech recognition percentage scores, there were no significant differences in speech recognition scores among the four groups of participants in this study (Thornton and Raffin, 1978). Mean unaided speech recognition scores for the *N*-back stimuli were 96% (SD = 2.7), 92% (SD = 4), 100% (SD = 0), and 98% (SD = 2) in quiet and 95% (SD = 1.4), 92% (SD = 3.3), 100% (SD = 0), and 96% (SD = 2.3) in background noise for the MA, YO, C-MA, and C-YO groups, respectively. Based on the 95% critical differences for speech recognition percentage scores, there were no significant differences in speech recognition scores among the four groups of participants in this study (Thornton and Raffin, 1978).

On average, MA participants used their hearing aids 12 h per day (SD = 5.5 h), and the YO participants used their hearing aids 11 h per day (SD = 6 h) based on hearing aid data log information. Aided speech recognition scores on the R-SPIN were 100% (SD = 0) in quiet and in background noise for the MA participants, and 100% (SD = 0) and 98% (SD = 1.3) in quiet and in background noise for the YO participants. Mean aided speech recognition scores for the *N*-back digits were 96% (SD = 2.6), and 95% (SD = 1.5) in quiet and 96% (SD = 1.6), 96% (SD = 1.2) in background noise for the MA and YO groups, respectively. Based on the 95% critical differences for speech recognition percentage scores, there were no significant difference between aided and unaided recognition of R-Spin sentences and *N*-back digits for either group of listeners (Thornton and Raffin, 1978).

### Listening Span Test

**FIGURE 2:**
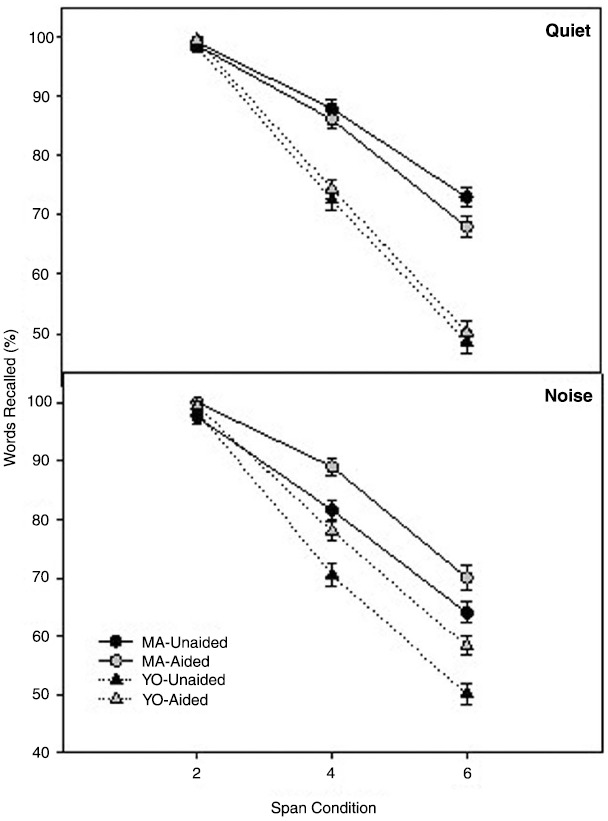
**Mean unaided and aided Listening Span test scores for the MA (circles) and YO (triangles) participants in quiet (top) and noise (bottom).** Error bars represent ± 1 SE.

Working memory function was assessed using the Listening Span test in quiet and in noise with and without hearing aids. Mean scores and standard errors of the mean on the Listening Span test in quiet and in noise, collapsed across context are shown in Figure 2. To compare differences in working memory across factors and participant groups, a 3 × 2 × 2 × 2 × 2 full factorial repeated measures analysis of variance (RMANOVA) was performed on the factors span (2 span, 4 span, 6 span), listening condition (quiet, noise), amplification (unaided, aided), context (low and high) and group (MA, YO). Greenhouse-Geisser corrections (Greenhouse and Geisser, 1959) were used to correct sphericity violations throughout the analyses where indicated. All *post hoc* comparisons were completed using the Bonferroni adjustment for multiple comparisons.

There was a significant two-way interaction of Span × Group [*F*(2, 20) = 9.6; *p* = 0.001; partial eta-squared = 0.50]. *Post hoc* analysis indicated significant group differences for the 4 and 6 span conditions but, not for the 2-span condition. YO participants scored significantly lower on the Listening Span test (i.e., poorer working memory performance) in the 4 span (*p* = 0.003) and 6 span (*p* = 0.001) conditions compared to the MA participants in both quiet and noise. There was also a significant two-way interaction between Listening Condition × Amplification [*F*(1, 21) = 4.8; *p* = 0.02; partial eta-squared = 0.20]. MA and YO participants scores on the Listening Span test were significantly (*p* < 0.001) higher (i.e., better working memory performance) with amplification in the 4 and 6 span conditions but, only in the background noise listening condition. Interestingly, while their performance was improved with hearing aids, the YO participants’ aided scores on the Listening Span Test approximated the unaided scores of the MA participants in the noisy listening condition.

**FIGURE 3:**
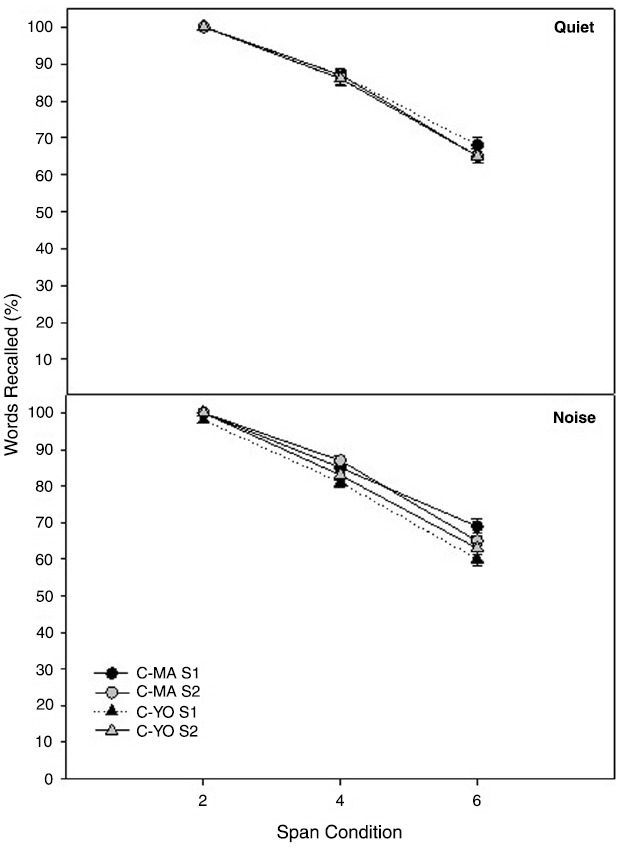
**Mean Listening Span test scores in quiet (top) and noise (bottom) for the two age-matched control groups [C-MA (circles) and C-YO (triangles)] for test sessions 1 and 2.** Error bars represent ± 1 SE.

Two age-matched control groups were used in this study to ensure significant changes in the experimental group were not a result of simply being re-tested on the Listening Span test over the 6 weeks. Mean Listening Span test scores for the MA and YO control participants in quiet and noise at their initial test session and at the second test session, which occurred 6 weeks later, are shown in Figure 3. To compare differences in scores on the Listening Span test over time for the MA and YO control groups, a 3 × 2 × 2 × 2 full factorial RMANOVA was performed on the factors span (2 span, 4 span, 6 span), listening condition (quiet, noise), test session (session 2, session 4), and group (C-MA, C-YO). There was a significant main effect of span [*F*(2, 26) = 48.29; *p* < 0.001; partial eta squared = 0.79]. Both groups of participants scored higher on the 2 span condition than the 4 and 6 span conditions (*p* < 0.001). All other main effects, two-way and three-way interactions were not significant (*P* > 0.05).

### *N*-back Test

**FIGURE 4:**
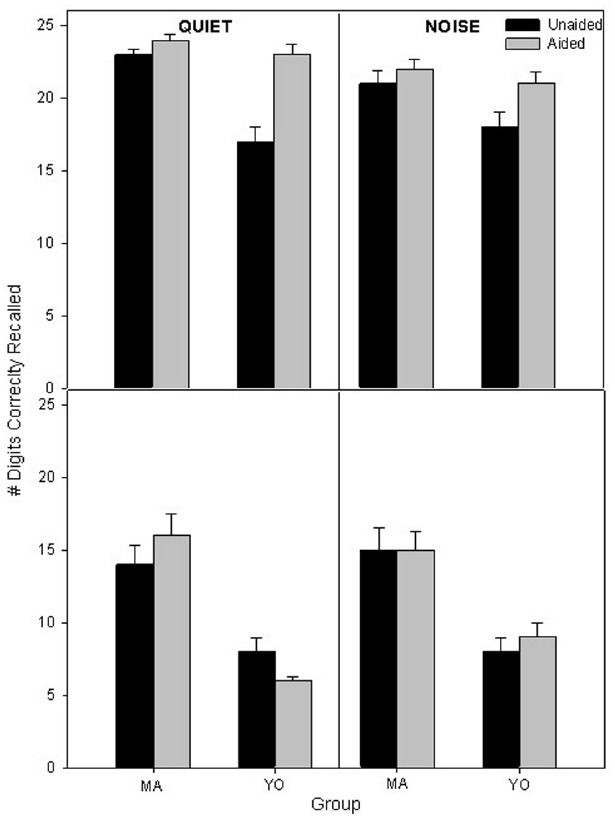
**Mean unaided and aided 1-back (top) and 2-back scores (bottom) in quiet and noise for the MA and YO participants.** Error bars represent ± 1 SE.

Participants’ mean unaided and aided scores on the auditory 1-back and 2-back in quiet and in background noise are displayed in Figure 4. To compare differences in performance on the auditory *n*-back across factors and participant groups, a 2 × 2 × 2 × 2 full factorial RMANOVA was performed on the factors back (1, 2) listening condition (quiet, noise), amplification (unaided, aided), and group (MA, YO). There was a significant three-way interaction of amplification × back × group [*F*(1, 19) = 7.2; *p* = 0.01; partial eta squared = 0.3]. *Post hoc* analysis indicated that the YO group scored significantly (*p* < 0.001) higher with hearing aids than without hearing aids in both the quiet and noisy listening conditions in the 1-back condition. However, there were no significant (*p* > 0.05) differences in 1-back scores for the MA participants in the quiet or background noise conditions with hearing aid use. Also, no significant (*P* > 0.05) differences were observed between aided and unaided performance in quiet and noise on the 2-back for either MA or YO participants.

**FIGURE 5:**
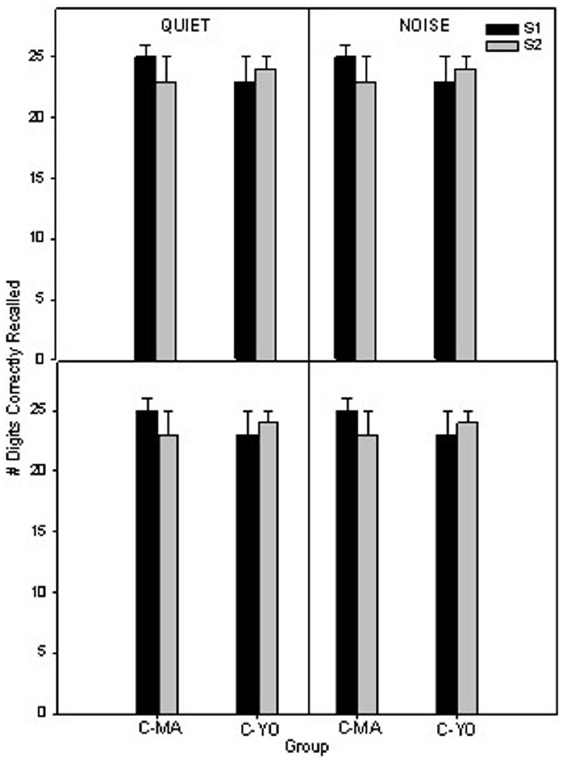
**Mean 1-back scores (top) and 2-back scores (bottom) in quiet and noise for the two age-matched control groups (C-MA and C-YO).** Error bars represent ± 1 SE.

In Figure 5 the mean 1-back and 2-back scores are shown for the MA and YO older control participants in quiet and noise at the initial test session and at a second test session which occurred 6 weeks later. To compare differences in scores on the auditory *n*-back over time for the MA and YO control groups, a 2 × 2 × 2 × 2 full factorial RMANOVA was performed on the factors listening condition (quiet, noise), test session (session 2, session 4), and group (C-MA, C-YO). There were no significant (*p* > 0.05) main effects, two-way or three-way interactions.

We also compared participants’ unaided auditory working memory performance on the *N*-back test in quiet and noise pre-fit and post-fit (6 weeks) to determine whether there was a cognitive transfer after using hearing aids for 6 weeks. A RMANOVA was performed on the within subject factors session (pre-fit, post-fit) and listening condition (quiet, noise) and the between subject factor group (MA, YO, C-MA, C-YO). There was no significant interaction between session and group [*F*(3, 33) = 1.25, *p* = 0.31, effect size = 0.1]. Therefore, it appears that the effect of hearing aids on working memory is more perceptual in that the benefit from amplification was directly related to the improved transfer of the signal and not cognitive transfer because wearing the hearing aids for 6 weeks did not change unaided working memory.

## Discussion

The purpose of this study was to assess the effect of hearing aids on auditory working memory function in MA and YO hearing impaired adults. The main finding of the current study was that MA and YO participants’ auditory working memory performance was significantly improved with hearing aid use. This finding is strengthened by the fact that we did not observe any significant changes in working memory performance on the Listening Span test or the auditory *n*-back test in either of the two age-matched control groups who were not fitted with hearing aids. Thus, the significant changes in the experimental group were not a result of simply being re-tested over time.

In this study, we specifically chose to measure the effects of hearing aids on working memory function because, numerous studies have reported on the importance of working memory ability for effective speech-communication in noise (Pichora-Fuller et al., 1995; Gatehouse et al., 2003; Humes et al., 2006; Vaughan et al., 2006; Akeroyd, 2008; Rudner et al., 2011; Besser et al., 2013), and how working memory ability declines with increasing age (Salthouse and Lichty, 1985; Park and Lee, 1999). In a review of twenty studies on speech recognition and cognitive abilities, Akeroyd (2008) found that while hearing sensitivity was the primary predictor of speech recognition performance, working memory capacity, as measured by the reading span test, was the second most important predictor. Long term memory is another factor that could influence listening under suboptimal conditions, e.g., in background noise, or with a hearing loss (Sörqvist and Rönnberg, 2012). Rudner et al. (2011) described how long term memory is used to help infer and construct the meaning of a target message by retrieving phonological, lexical, and semantic representations from an individual’s long term memory. However, the current study focused on measuring auditory working memory.

In the current study, both the MA and YO participants’ Listening Span test scores were significantly higher with hearing aid use. Although the YO participants working memory performance was improved with hearing aids, their performance never achieved the level to that of the MA group. Interestingly, the YO participants *aided* scores on the reading span test approximated the *unaided* scores of the MA group. This suggests amplification may reduce the confounding effect of hearing loss on apparent early age-related decline in auditory working memory function.

Significant improvements in working memory performance with hearing aids for both MA and YO groups on the auditory Reading Span test were only evident when memory performance was tested in background noise, even though their speech recognition scores were excellent in both quiet and noise. This result largely supports the *effortfulness hypothesis*: the theory that the extra effort that a hearing-impaired listener must expend to successfully understand speech comes at the cost of cognitive processing resources that might otherwise be available for encoding the speech content in memory (Rabbitt, 1968; Kahneman, 1973; Wingfield et al., 2005; Tun et al., 2009). In other words, speech understanding in everyday life is influenced by both bottom-up and top-down cognitive functions that moderate the processing of auditory information (Gatehouse et al., 2003; Humes et al., 2006; Vaughan et al., 2006; Desjardins and Doherty, 2013, 2014). Because speech contains redundant information a hearing-impaired individual can cognitively compensate by “filling in” missed information. Thus, top-down cognitive compensations can effectively mask a peripheral hearing loss and help the hearing impaired listener function more effectively in everyday listening situations (e.g., Tun et al., 2009; Gosselin and Gagne, 2011; Rudner et al., 2011; Desjardins and Doherty, 2013; Rönnberg et al., 2013; Zekveld et al., 2013). For example, in the present study the cognitive demand of the Listening Span Test was greater in noise than in quiet, and therefore required listeners to use more cognitive resources (Murphy et al., 1999; Desjardins and Doherty, 2013, 2014). It is likely that we did not observe significant improvements in working memory scores with hearing aids in the quiet listening condition because the cognitive load did not exceed the individual’s cognitive capacity. This result is consistent with a recent study by Mishra et al. (2014) that found residual cognitive capacity, termed Cognitive Spare Capacity, was not reduced in an older group of hearing impaired adults when listening conditions were optimal, but was reduced when the speech was presented in background noise.

Hearing aid use also improved participants’ performance on the auditory 1-back task but, no improvements were observed on the 2-back task. On the 1-back only improvements in task scores were observed for the YO group. Interestingly, unlike their performance on the Listening Span Test, the YO participants’ aided scores on the 1-back were significantly higher in both quiet and in background noise compared to their unaided scores. It is not too surprising that we observed a different pattern of results on the auditory 1-back compared to the Listening Span test. Several studies which have measured the convergent validity of the *n*-back task with other measures of working memory (see Kane et al., 2007) have largely revealed weak or modest correlations between individuals’ performance on the *n*-back task and performance on other standard, accepted assessments of working memory (Kane et al., 2007; Jaeggi et al., 2010). This is because, performance on the *n*-back task seems to be more closely correlated with performance on measures of fluid intelligence than it is with performance on other measures of working memory (Jaeggi et al., 2010). This is interesting because the 1-back, although having a low cognitive load, proved to be a more sensitive measure for assessing hearing aid use in our older group of adults, as significant differences with amplification were observed in both quiet and noisy listening situations. However, it was not sensitive to changes in auditory working memory function with hearing aid use in the MA participants. It is likely that we did not see significant improvements with amplification on the 1-back in the MA group because of a ceiling effect on the task, as their unaided performance was already near excellent. It is somewhat difficult to interpret why there was no improvement with amplification on the 2-back task for either the MA or YO participants. Perhaps the perceptual benefit of amplification could not improve performance on a task with such a high cognitive load.

If the benefit from amplification on the 1-back test was more of a cognitive transfer effect, then the participants’ *unaided* working memory performance should have improved after wearing the hearing aids for 6 weeks, which did not occur. Therefore, the effects of amplification was more perceptual (immediate effect on encoding of working memory) than a cognitive transfer (long-term). Another way to measure this would have been to measure aided and unaided performance on the cognitive tests at weeks 1 and 6, and assess if the amount of hearing aid benefit increased over time. However, we did not obtain aided scores on week 1 because this was part of a larger longitudinal hearing aid study, which did not include aided testing at week 1. Regardless, results from the present study indicate that some type of frequency shaping/amplification should be used when testing auditory working memory in hearing-impaired adults, even with a mild degree of hearing loss, to reduce the potential negative effect a degraded peripheral representation of the signal could have on cognitive test scores.

Using hearing aids in the early stages of age-related hearing loss, even when hearing loss is mild, can improve performance on auditory working memory tests in quiet and in background noise. The majority of MA and YO adults are still in the workforce, and although they may be able to “get by” without a hearing aid, it is important to consider the impact of their hearing loss on their working memory function. Although results from this study indicate wearing hearing aids can have a positive impact on working memory performance, future research should investigate if using hearing aids during the earlier stages of age-related hearing loss can reduce or even prevent some of the perceptual changes that result from auditory deprivation (Thai-Van et al., 2010).

### Conflict of Interest Statement

The authors declare that the research was conducted in the absence of any commercial or financial relationships that could be construed as a potential conflict of interest.
